# Nanocurcumin in myocardial infarction therapy: emerging trends and future directions

**DOI:** 10.3389/fbioe.2024.1511331

**Published:** 2025-01-08

**Authors:** Mei Lv, Qing Sun, Yilin Yu, Jinwei Bao

**Affiliations:** ^1^ General Medicine Department, Yantaishan Hospital, Yantai, Shandong, China; ^2^ Department of Cardiology, Yantaishan Hospital, Yantai, Shandong, China; ^3^ Preventive medicine, Shandong University, Jinan, Shandong, China

**Keywords:** myocardial infarction, nanotechnology, cardioprotective, apoptosis, curcumin

## Abstract

Myocardial infarction (MI) is the leading cause of morbidity and mortality worldwide. Curcumin has been observed to significantly reduce pathological processes associated with MI. Its clinical application is limited due to its low bioavailability, rapid degradation, and poor solubility. Advancements in nanotechnology can be used to enhance its therapeutic potentials in MI. Curcumin nano-formulation enhances its solubility, stability, and bioavailability, allowing more precise delivery to ischemic cardiac tissue. Curcumin nanoparticles have been observed to successfully reduce infarct size, maintain heart function by modulating essential molecular pathways in MI. Its liposomal formulations provide sustained release and higher tissue penetration with improved pharmacokinetics and enhanced therapeutic efficacy. Preclinical studies revealed that nanocurcumin drastically lower oxidative stress indicators, inflammatory cytokines, and cardiac damage. Micelles composed of polymers have demonstrated high biocompatibility and targeting capabilities with increased cardio-protective effects. Research and clinical trials are essential for comprehensive analysis and efficacy of curcumin-based nano-therapeutics in cardiovascular condition and lowering risk of MI.

## 1 Introduction

Dried turmeric powder from the rhizome of *Curcuma longa* has been extensively utilized for decades in some parts of the world. It has various biological functions, including anti-inflammatory, anti-aging, anti-cancer, anti-arthritic, anti-growth, anti-atherosclerotic, wound healing, antimicrobial, and memory-boosting properties (Aggarwal et al., 2013). Curcumin (Cur) has been investigated for its pleiotropic actions in myocardial infarction (MI) and explored as a promising therapeutic candidate (Aggarwal et al., 2007). Cur has also indicated cardioprotective potentials against arrhythmia, hypertrophic cardiomyopathy, diabetic cardiomyopathy, myocardial ischemia injury, and cardiotoxicity induced by doxorubicin (Salehi et al., 2020). Literature suggests that these effects arise from curcumin’s capacity to mitigate inflammation, apoptosis, and oxidative stress (Jiang et al., 2017). It has long been employed to treat inflammatory illnesses, jaundice, coughs, colds, and hepatic problems (Aggarwal and Harikumar, 2009). It has also been comprehensively studied and employed in various pharmaceutical, food, and textile industries (Boroumand et al., 2018).

The primary active constituent isolated from the turmeric rhizomes is a polyphenolic molecule, curcumin (1,7-bis(4-hydroxy-3-methoxyphenyl)-1,6-heptadiene-3,5-dione). Cummin’s chemical structure gives it potent phytochemical properties with anti-inflammatory characteristics. It can interact with multiple inflammatory pathways, making it a promising option for preclinical and clinical use (Boroumand et al., 2018). Milobedeska and Lampe *et al.* clarified and synthesized curcumin’s structure (Gupta et al., 2012). Structurally similar chemicals 17% dimethoxycurcumin and 3% bisdemethoxycurcumin are also present in curcumin preparations (Sharma and Sharma, 2022). Several studies have indicated that its anti-inflammatory characteristics result in decreased expression of cyclooxygenase-2, IL-6, prostaglandin E2, and tumor necrosis factor-α (TNF-α) genes (Boroumand et al., 2018). In addition, Cur can scavenge reactive oxygen species (ROS), inhibit xanthine oxidase and lipid peroxidation, as well as enhance the activity of antioxidant enzymes such as glutathione peroxidase (GPX), superoxide dismutase (SOD), and catalase (Kukongviriyapan et al., 2016).

A significant challenge in the administration of curcumin is its poor solubility in water, with estimated solubility of 0.6 μg/mL at pH 7.4, and 11 ng/mL in aqueous buffer at pH 5. As a result, 60%–70% of the oral medicine is not absorbed (Tønnesen et al., 2002). Cur has hydrophobic properties with octanol-water coefficient partition value of 3.29, which provides the molecules with acceptable permeability across membranes in biological systems. Biopharmaceutical Classification System (BCS) has categorized Cur as a class II drug, indicating low solubility and high permeability (Wan et al., 2012). Light enhances Cur instability, demonstrating disintegration within the solid state and solution with UV/visible radiation (Shen and Ji, 2012). The primary challenge for optimizing curcumin’s biological activity is its low bioavailability due to poor aqueous solubility, leading to its quick metabolism, excretion, and decreased uptake in tissues and serum (Anand et al., 2007).

Drug delivery for MI faces significant challenges, particularly with targeted and nanotherapeutic treatments. Key physiological barriers like blood clots, extracellular matrix, and vascular endothelium, hinder drug penetration into damaged cardiac tissue. Additionally, post-MI events characterized by inflammation, scar formation, hypoxia, and oxidative stress, further complicate drug efficacy and retention. Pharmacokinetics limitations like poor absorption and rapid clearance, reduce drug availability at the site of injury in MI (Karam et al., 2022). Non-targeted drugs cause systemic toxicity. To address these challenges, researchers are exploring innovative approaches like stimuli-responsive carriers, targeted nanoparticles, and controlled-release biomaterials to enhance drug delivery, improve specificity, increase bioavailability, and achieve better therapeutic outcomes for MI treatment (George et al., 2022).

Encapsulation of natural products and their derivatives has indicated significant benefits including less systemic adverse effects, enhanced biosafety, excellent drug dissolution and bioavailability, extended duration of circulation, and controlled accumulation in targeted organs (Taghipour et al., 2019). Nano-medicine is a novel approach with reduced incidence of pharmaceutical adverse effects and improved efficacy (Rahimi et al., 2016). Several studies have investigated curcumin nano-formulations for their delivery to improve its therapeutic efficacy for various disorders like cancer (Yallapu et al., 2012), neurodegenerative disorders (Rakotoarisoa and Angelova, 2018), wound healing (Hussain et al., 2017), diabetes (Maradana et al., 2013), and inflammatory diseases (Mahmood et al., 2015). Various nanotechnology-based drug delivery systems have been employed for curcumin administration, including polymeric, carbon nanotubes, solid lipid, nano gel, dendrimers micelle, cyclodextrin inclusion complexes, noisome, nano emulsion, exosome, nanosuspensions, nanocrystals, and mesoporous silica nanoparticles. These platforms are utilized in tissue engineering to improve the therapeutic efficacy of Cur (Yallapu et al., 2015; Ahangari et al., 2019). Nano-medicine compositions have several advantages, including enhanced circulation lifetime, pharmaceutical kinetics, permeability, and capacity to modify physiological barriers, hydrophobic drug solubility in water, and the ability to design controlled release formulations (Naksuriya et al., 2014). The limiting factors for Cur application can be addressed by increasing its bioavailability, shielding it from metabolism and degradation, and increasing its targeting ability. The advantages of nano-mediated drug delivery include increased bioactivity and bioavailability *via* surface modifications, decreased particle size, and nano-carrier entrapping (Gera et al., 2017).

Herein, we discuss the application of nanotechnology to increase the delivery and efficacy of Cur and its derivatives for MI. There are many challenges associated with the clinical use of nanocarriers. Their scalability, and *in vivo* biocompatibility are the main challenges associated with these novel systems. Advances in multi-functional nanocarriers that co-deliver Cur with other therapeutic agents (anti-inflammatory drugs or growth factors) could lead to the development of a multi-aspect MI pathology targeted approach to improve treatment outcomes. Finally, despite challenges, nanotechnology offers great promise in optimizing curcumin-based therapies for MI. These advanced delivery systems deliver effective, targeted, and safe therapeutic options to patients with cardiovascular diseases.

## 2 Curcumin intracellular localization and hormetic effects in myocardial infarction

The intracellular behavior of curcumin, including delivery, concentration, and localization are critical aspects in determining curcumin’s therapeutic efficacy in disease conditions including cardiovascular diseases. Low doses of drugs and other stressors are often innocuous and generate an adaptive stress response that increases resistance to greater concentrations (a phenomenon known as hormesis) (Rainey et al., 2015). Curcumin has hormetic properties as its effects are highly dose-dependent. Therefore, understanding of intracellular dynamics of Cur action for optimizing its clinical use is very important (Rainey et al., 2020). Nanotechnology offers promise for the improvement of Cur bioavailability, and provision of safer and effective treatment regimens for cardiovascular diseases (Sharma and Sharma, 2022). Despite extensive research, delivery of Cur to and intracellular concentration in cellular compartments remains unclear. Since its therapeutic efficacy is extremely dependent on a specific subcellular localization and intracellular concentration which determines whether curcumin acts as a cytoprotective agent or results in apoptosis. Optimizing curcumin’s potential for the treatment of cardiovascular diseases depends on understanding of these intricately cellular behaviors (Sala de Oyanguren et al., 2020).

Following internalization, Cur is distributed to different key organelles including mitochondria, lysosomes and endoplasmic reticulum. These organelles have important role in cellular homeostasis, and the capacity of curcumin to target and accumulate in these organelles is directly related to therapeutic outcomes (Rainey et al., 2020). Cell death and tissue damage produced by ischemia-reperfusion injury are mediated by mitochondrial dysfunction (Jurcau and Ardelean, 2022). Curcumin’s primary mitoprotective effect is its antioxidant activity. It neutralizes reactive oxygen species, enables to mitigate oxidative stress, and protects mitochondrial integrity. It also effects mitochondrial dynamics including fission, fusion, and biogenesis to maintain the function of mitochondria and support cell survival under stress conditions (de Oliveira et al., 2016). Specifically, these actions are important in CVD where mitochondrial dysfunction is an important component of the pathophysiology of ischemic injury and cardiomyocyte death (Chistiakov et al., 2018). The lysosome is an important organelle in cellular waste disposal and homeostasis (Shen et al., 2023). In low concentrations, curcumin promotes the degradation of damaged organelles and proteins by enhancing autophagic flux. It stimulates the recycling of cellular components to promote cell survival. At higher concentrations it induces lysosomal membrane permeabilization (LMP), resulting in the release of lysosomal enzymes into the cytoplasm, triggering the cytotoxic cell death (Shen et al., 2023). Protein folding, lipid synthesis, and calcium regulation are the main roles of endoplasmic reticulum. The ER activates unfolded protein (UPR) response under stress conditions like ischemia or oxidative injury and maintains cellular homeostasis. Curcumin modulates ER stress responses. It acts as a chaperone that helps proteins fold and prevent them from accumulating as misfolded proteins. By relieving ER stress, curcumin inhibits cellular apoptosis and contributes to the support of cellular function in ischemic heart tissue (Amen et al., 2019; Shakeri et al., 2019; Moustapha et al., 2015).

## 3 Therapeutic potential of curcumin in myocardial infarction

Curcumin is a promising therapeutic agent for MI due to its ability to address underlying cellular damage caused by inflammation and oxidative stress, unlike conventional drugs such as beta-blockers, ACE inhibitors, and anticoagulants, which mainly manage symptoms (Xiao et al., 2016). Cur inhibits key inflammatory pathways like COX-2 and NF- κB. It also possesses potent antioxidant properties that neutralize free radicals, reduce oxidative stress, and offer a more comprehensive approach to treatment in contrast to conventional standard practices (Khezri et al., 2021). Despite having poor bioavailability in its natural form, Cur is often well tolerated compared to standard medicines that frequently have substantial adverse effects such as hypotension, bradycardia, or bleeding hazards. To overcome this challenge, advances in nanotechnology lead to nano formulations that enhance the therapeutic efficacy of Cur by improving its absorption and retention in the body. In addition, Cur has a multi-targeted activity in MI therapy, boosting angiogenesis and inhibiting fibrosis which are essential for long-term heart repair and function (Sadeghi et al., 2023).

**FIGURE 1:**
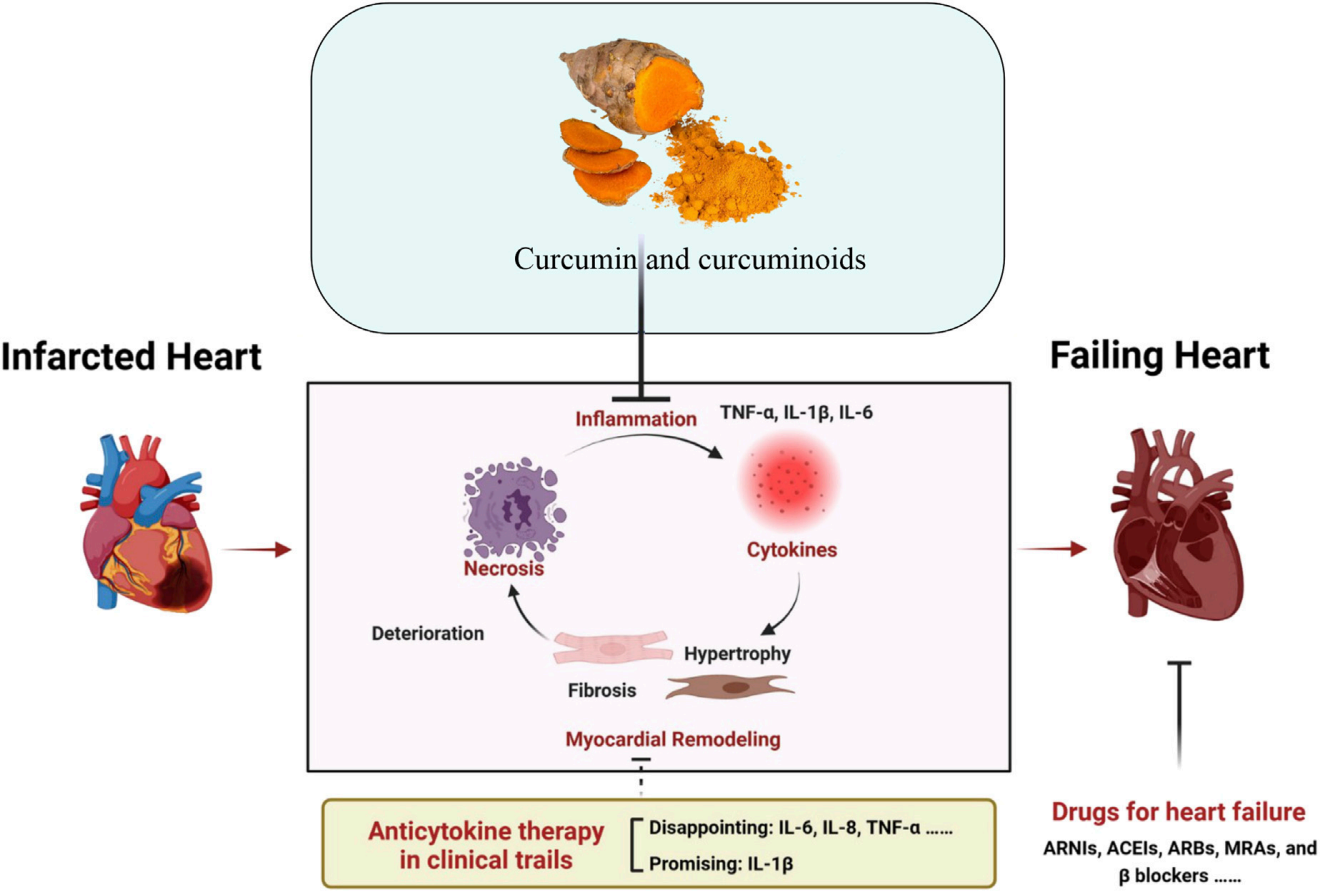
Schematic illustration of the anti-inflammatory role of curcumin and approved standard treatment drugs for MI and Heart failure. Adapted with slight modification from reference (Zhang et al., 2023) under a Creative Commons Attribution 4.0 International License (http://creativecommons.org/licenses/by/4.0/).

The present drugs used in clinical settings for MI are intended to re-establish circulation and reduce cardiac damage and further adverse cardiovascular events. The use of antiplatelet agents such as aspirin and P2Y12 inhibitors (clopidogrel, ticagrelor) are employed to inhibit platelet aggregation which is essential in preventing blood clots. Alteplase (tPA) and streptokinase are thrombolytics used for dissolving clots early in an MI, especially when percutaneous coronary intervention (PCI) is not readily available (Hesari et al., 2021). Heparin is utilized to prevent the formation of new blood clots. Beta-blockers like metoprolol decrease the heart rate and lower blood pressure, reducing the heart’s workload. ACE inhibitors, such as Ramipril and ARBs (losartan) help to relax blood vessels, improve heart function, and reduce high blood pressure. Statins, like atorvastatin, stabilize arterial plaques and reduce cholesterol, which lowers the risk of further MI (Banerjee, 2020). By widening blood arteries, nitrates such as nitroglycerin reduce chest pain, while aldosterone antagonist spironolactone helps in the management of heart failure and inhibits cardiac remodeling following MI (Grujić-Milanović et al., 2023). These medications are frequently combined and serve as the cornerstone of MI treatment to enhance patient outcomes and survival. It has been observed that MI can cause endothelial and microvascular damage and even cellular death (Duehrkop and Rieben, 2014). Curcumin offers a holistic treatment option for the treatment of MI due to its cardioprotective effects, inhibition of myocardial fibrosis, ventricular remodeling after acute attack and prevents the progression of heart failure after MI (Li et al., 2023). The beneficial effects of curcumin and curcuminoids have been assessed in clinical settings. As an example, in a study, curcuminoids treatment significantly reduced the plasma malondialdehyde (MDA) and C-reactive proteins and reduced the incidence of MI in patients who underwent coronary artery bypass graft surgery (CABG) (Wongcharoen et al., 2012). In a pre-clinical rat MI model where MI was induced with left anterior descending (LAD) coronary artery ligation, curcumin through the inhibition of p38 MAPK and JNK and activation of RISK/GSK-3β protected against myocardial ischemia/reperfusion injury (Jeong et al., 2012). The therapeutic role of Curcumin and small molecular conventional drugs has been shown below in Figure 1.

### 3.1 Oxidative stress and myocardial infarction

**FIGURE 2:**
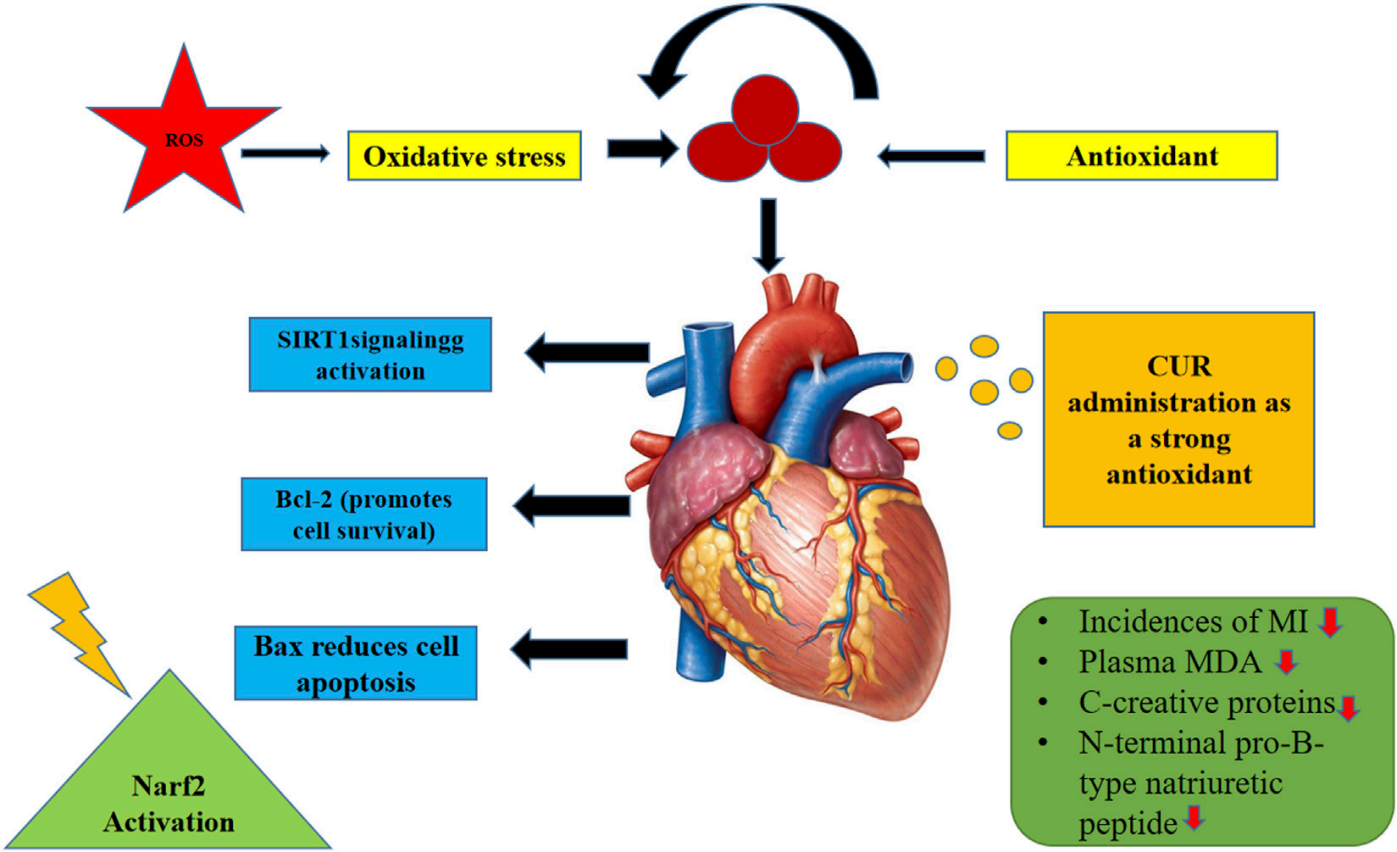
Schematic diagram illustrating the role of Curcumin in mitigating oxidative stress and MI.

Oxidative stress results after an imbalance between the antioxidant and oxidant systems. Under normal physiological state, cells contain low oxidants and oxygen radical concentrations, which modulate cellular equilibrium, differentiation, mitosis, survival, and signaling (Bagheri et al., 2016). Oxidative stress has been associated with genesis and etiology of various disorders, including ischemic myocardial injury (Yang Y. et al., 2013). Recent studies have validated that Cur has strong antioxidant activity which can prevent or reduce ischemia-reperfusion (I-R) injury. Cur induces silent information regulator 1 (SIRT1) signaling, which promotes the overexpression of Bcl-2 and the downregulation of Bax, thereby mitigating mitochondrial insufficiency after myocardial I-R (Zhang et al., 2024). Cur may also maintain the redox potential of the mitochondria by markedly enhancing the activity of the superoxide dismutase (SOD) enzyme and reducing the production of MDA and hydrogen peroxide within the mitochondria (Yang Y. et al., 2013). Furthermore, it also promotes the activation of nuclear factor erythroid-derived 2 (Nrf2)- a nuclear basic leucine zipper transcription factor that belongs to the NF-E2 family, and controls the expression of several genes involved in pro-oxidative stimuli detoxification (Fisher et al., 2007). A clinical experiment investigated the beneficial effect of curcuminoids in reducing myocardial I-R damage after CABG. Curcuminoids (4 g/day) were supplemented beginning 3 days following CABG and before 5 days. The results reported a significantly lower incidence (17%) of in-hospital MI in the curcuminoids group as compared to placebo. Plasma concentrations of MDA (biochemical indicator of oxidative stress and lipid peroxidation), C-reactive protein (diagnostic of systemic inflammation), and N-terminal pro-B-type natriuretic peptide were reduced after curcuminoids consumption (Wongcharoen et al., 2012). Schematic diagram illustrating the role of Cur in mitigating oxidative stress and MI is depicted in Figure 2.

### 3.2 Apoptosis and myocardial infarction

Apoptosis has been extensively studied and occurs naturally in multicellular organisms. However, specific diseases can trigger apoptotic pathways, leading to unintended cell necrosis. For example, cardiac I-R initiates the apoptotic pathways, leading to cardiomyocyte death (Wang Y. et al., 2014). Duan *et al.* investigated rat hearts and indicated that curcumin protects against the harmful effects of myocardial I-R (Duan et al., 2012). Knockdown of the JAK kinase-specific inhibitor AG490 revealed that curcumin alleviated the I–R damage caused by the JAK2–STAT3 signaling pathway activation, which was reflected in the overexpression of Bcl-2 and downregulation of caspase-3 (Duan et al., 2012). In addition, Jeong *et al.* revealed that curcumin inhibits numerous prosurvival kinases to protect against regional myocardial I-R damage (Jeong et al., 2012). Curcumin has been observed to significantly enhance the antiapoptotic cascade kinases glycogen synthase kinase-3β (GSK-3β), Akt, phosphoinositide 3-kinase (PI3K), and extracellular signal-regulated kinase 1/2 (ERK1/2) were all phosphorylated. p38 MAPKS is one of the most significant signaling pathways that regulate cellular stressors and diverse pathological states, such as cardiac I-R (Jeong et al., 2012) and cell death. Cur therapy decreased p38 MAPK and c-Jun NH2-terminal protein kinase (JNK) phosphorylation levels (Wang J. et al., 2023).

### 3.3 Autophagy and myocardial infarction

Autophagy is essential for cardiomyocytes to maintain cellular homeostasis (Huang et al., 2015). During physiological conditions, the basal autophagy level in the cardiovascular system is sufficient to protect cells from energy constraints and eliminate damaged organelles and superfluous proteins. Natural components in food, including Cur regulate autophagy by inducing cardioprotective effects which shields mouse cardiomyocytes from oxidative stress (Salabei and Conklin, 2013; Han et al., 2012). Several studies indicated protective autophagy is associated with the activation of the early myocardial ischemia stages and the AMPK pathway (Yang K. et al., 2013). Cur has been observed to prevent I-R damage in H9c2 myocytes by inhibiting increased autophagy and apoptosis, and downregulating the expression of SIRT1, Beclin-1, Bax, BNIP3, and Bcl-2 (Huang et al., 2015).

### 3.4 Inflammation and myocardial infarction

Complex processes modulate inflammatory reactions and are essential components of host defense. Leukocytes infiltrate the infarcted myocardial area, triggering inflammatory response (Biswas, 2016). However, excessive inflammatory response can damage heart perfusion (Pashkow, 2011). A study indicated that NF-κB signaling is an essential proinflammatory signaling route implicated in cardiac I-R damage. Fiorillo *et al.* revealed that besides antioxidant protective mechanism, Cur promotes its cardioprotective effects *via* multiple pathways including NF-κB and JNK pathways (Fiorillo et al., 2008). Cur has been observed to reduce pathological alterations associated with I-R used before or after I-R therapy. Kim *et al.* showed that pretreatment with Cur (300 mg/kg/day) for 7 days in SD rats decreased the expressions of TLR2 and MCP-1 in cardiomyocytes after I-R damage, inhibited activation of TNF-α, reduced fibrotic response, and inhibited macrophage infiltration (CD68) in cardiac tissues (Kim et al., 2012). The summary of the main molecular mechanisms of curcumin in MI is given in Table 1.

**TABLE 1:** General molecular target of curcumin in myocardial ischemia perfusion injury.

Action	Molecular mechanism	References
Oxidative stress	Curcumin’s potential mechanism for protecting against cellular oxidative stress includes inhibiting the synthesis of ROS and scavenging them, protecting the mitochondrial structure, particularly mitochondrial redox capacity	Wang et al. (2013), Yang et al. (2013c), Cox et al. (2022), Yan et al. (2023)
Apoptosis	Curcumin exerts anti-apoptotic effects by inhibiting the synthesis of pro-apoptotic proteins, specifically Bcl-2-associated X protein (Bax) and the protein caspase. Furthermore, this effect might be mediated by the kinase 2 and signal transducer and activator of transcription 3 (JAK2/STAT3) signaling pathways	Jeong et al. (2012), Abolfazli et al. (2024), Yang et al. (2023), Abbaszadeh et al. (2020)
Inflammation	Curcumin inhibits the expression of Early Growth Response-1 (Egr-1) expression in the ischemic heart, thereby downregulating proinflammatory cytokine levels. This is associated with decreased levels of TNF-A and IL-6, fewer infarcts, and reduced ischemic injury	Li et al. (2023), Wang et al. (2014b)

## 4 Current trends of nanotechnology-based curcumin formulations in myocardial infarction

Nano-technology has significantly improved the therapeutic efficiency of many medications and has provided novel and potent alternatives for the treatment of various chronic illnesses. Their small particle size and large surface area enable them to change the pharmacokinetic characteristics of bioactive compounds with low bioavailability (Demetzos and Pippa, 2014). Nano-systems have increased drug targeting, improved drug distribution, enhanced bio-distribution, and protected the drug from endogenous (enzymes, acid media, and first-pass effect) and exogenous (heat and light) degradation effects (Watkins et al., 2015). Because of the Cur bioactive and therapeutic properties, it has received significant attention from the scientific community (Gupta et al., 2013). In recent studies, encapsulation of curcumin with nanoparticles (liposomes, solid lipid nanoparticles, and polymeric nanoparticles) has been shown to increase curcumin solubility, stability, and pharmacokinetic profile. Controlled and targeted drug delivery with these Nano formulations reduces systemic toxicity while increasing the bioactivity of curcumin.

Curcumin-loaded nanocarriers have been shown to effectively target cancer cells, induce apoptosis, reduce tumor growth, and minimize adverse effects (Bertoncini-Silva et al., 2024). Several recent studies emphasize that particle size, in particular, is important to improve curcumin’s pharmacokinetic performance. Cellular uptake, bio-distribution and tissue penetration are improved by nanoparticles of 10–200 nm. In particular, nanoparticles in the 50–100 nm range take advantage of the enhanced permeability and retention (EPR) effect to improve tumor tissue accumulation and thus therapeutic efficacy in cancer treatment. In addition to size, preserving curcumin’s bioactivity during systemic circulation requires that nanoformulations are stable (Jacob et al., 2024). Advancements in liposomes, solid lipid nanoparticles (SLNs), and polymeric nanoparticles have provided recent protection against enzymatic, acidic, and oxidative degradation of curcumin. For example, SLNs greatly increase curcumin solubility and provide sustained drug release, thereby improving curcumin’s therapeutic efficacy *in vivo* (Subroto et al., 2023). Additionally, the targeting ligand-functionalized nanoparticles enable specific delivery to the cancer cells or the inflamed tissues while ablating the chances of off-target effects and systemic toxicity (Luo et al., 2023).

Recent studies revealed that nano-formulations are increasingly being developed to improve Cur bioavailability, circulation time, and targeted delivery to ischemic tissues. This includes leveraging processes such as pH-sensitive responsive systems and receptor-mediated endocytosis (Sadeghi et al., 2016). Furthermore, efforts have been made to combine Cur with other bioactive agents, enhancing its synergistic effects in multi-drug therapies.

### 4.1 Development of liposomal curcumin nanoparticles

Liposomes are spherical vesicles that mimic cell membranes, made up of one or more phospholipid bilayers (Bozzuto and Molinari, 2015). Nanoparticles encapsulating liposomes are employed in nano-drug formulations. Liposomes have many advantages including enhanced solubility, controlled dispersion, ease of manufacturing, high stability, minimal toxicity, and targeting ability of distinct cells (Tiwari et al., 2012). Several studies indicated that liposomal curcumin provides the best delivery system for treating heart failure (Jiang et al., 2017; Çağdaş et al., 2014). Recently, liposomes have been synthesized to co-deliver atorvastatin calcium and Cur to treat atherosclerosis (Li et al., 2019). Cur as a safe adjunct, has been found to reduce the harmful effects of atorvastatin calcium while enhancing its anti-atherosclerotic properties. To enhance the effectiveness of this treatment, specific ligands have been integrated into the liposome membrane to facilitate targeted delivery. The combined action of these two active ingredients leads to a reduction in pro-inflammatory factors and the formation of atherosclerotic plaques (Li et al., 2019). Moreover, liposomes co-encapsulating resveratrol and Cur have indicated increased encapsulation efficiency, reduced polydispersity index (PDI), and smaller particle size (Huang et al., 2019). Consistently, the literature has indicated that curcumin loaded liposomes possess anti-inflammatory, antioxidant, and cardio-protective properties (Salehi et al., 2020; Basnet et al., 2012).

**FIGURE 3:**
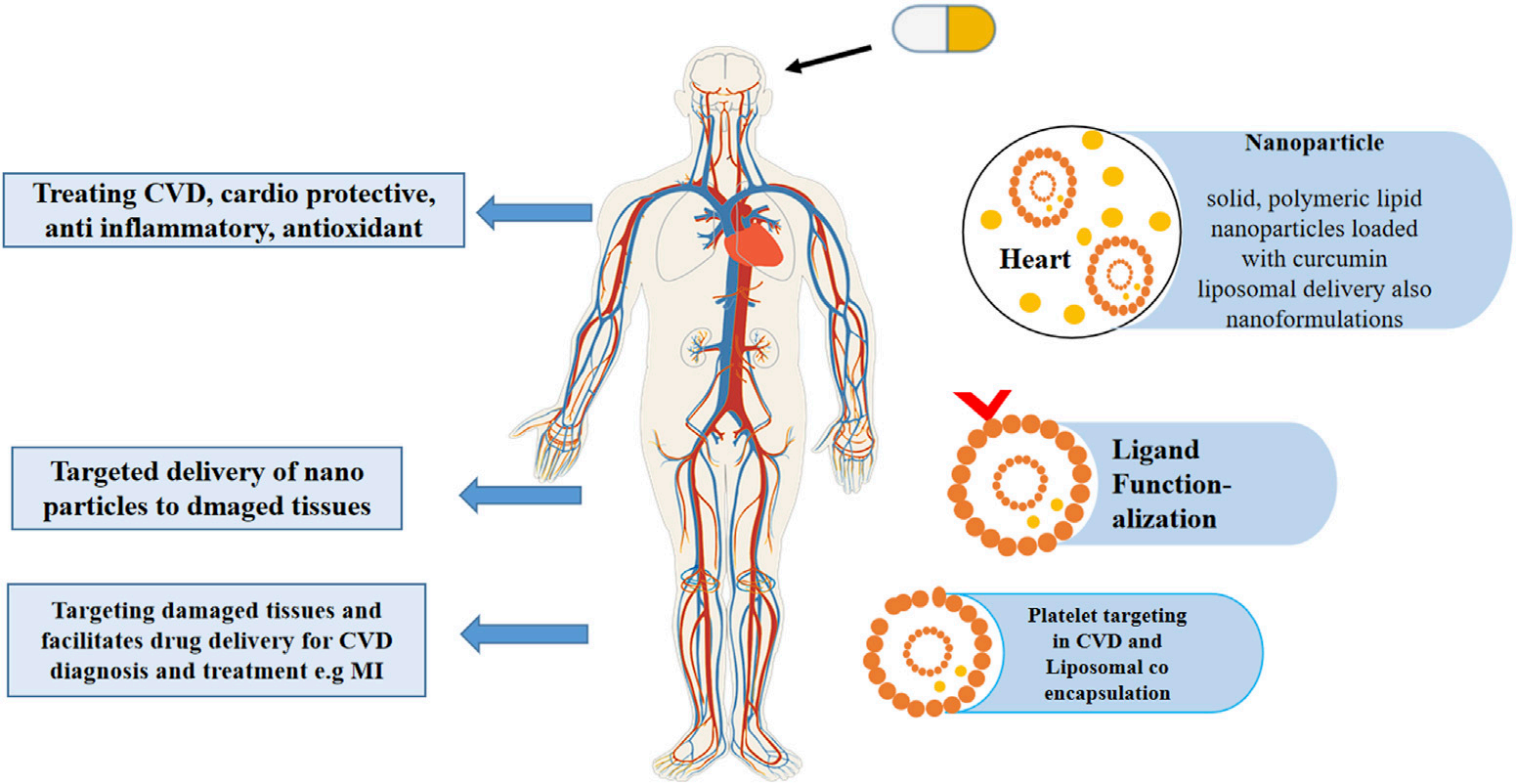
This schematic illustration represents how liposomes act as advanced drug carriers for treating various cardiovascular conditions, highlighting the co-delivery of curcumin and atorvastatin and the targeting of specific tissues through functionalized vesicles.

Liposomal scaffolds are an effective tool for platelet targeting in CVD diagnosis and treatment. It has shown that the natural peptides on the liposomal surface can help target damaged or diseased tissues and facilitate the transfer of active pharmaceutical drugs. Several research studies have indicated that atherosclerosis, MI, aneurysms, angiogenesis, and other CVD outcomes support nano-technology in theranostics (Pala et al., 2021). Figure 3 represents how liposomes act as advanced drug carriers for treating various cardiovascular conditions, highlighting the co-delivery of curcumin and atorvastatin and the targeting of specific tissues through functionalized vesicles.

### 4.2 Development of polymeric encapsulated curcumin nanoparticles

Solid lipid, polymer, gold, magnetic, and albumin-based nanoparticles have been used to increase the therapeutic applications of curcumin (Danhier et al., 2012). Biocompatible polymeric nanoparticles can stay in the bloodstream for long periods. Various natural and synthetic polymers are employed to form nanoparticles such as polyvinyl alcohol (PVA), polyethylene glycol, silk fibroin, hydrophobic compounds modified starch, and low-density chitosan (Fredenberg et al., 2011). Boarescu *et al.* investigated the effect of pretreating rats with free Cur and CurNP to treat isoproterenol-induced MI. Doses of 100, 150, and 200 mg/kg were administered in rats for 15 days, and on the 13th day, MI was induced (Boarescu et al., 2019a). Blood samples were collected during studies to assess the antioxidant, cardio-protective, and anti-inflammatory properties of the CCNP by measuring the oxidative stress indices, as well as several enzymes (CK and CK-MB). It was observed that all doses of Cur and CCNP prevent CK-MB outburst from cardiomyocytes, suggesting its cardio-protective effect. A study indicated that rats orally administered with nanoparticles of hydroxyapatite (HAPNPs) had cardiac toxicity (Yousef et al., 2021). This was evident from the increased serum MI markers, such as CK-MB, changes in routine blood factors, increased serum inflammatory markers, P53 expression (apoptotic protein), and interleukin-6 (IL-6), as well as decreased glutathione and cardiac antioxidant enzyme levels. Significant histopathological and histochemical changes were observed in the rats’ hearts, along with lipid peroxidation and nitric oxide. However, the co-administration of Cur and chitosan nanoparticles effectively alleviated these changes (Mosa et al., 2021).

### 4.3 Development of solid lipid nanoparticles of curcumin

Solid lipid nanoparticles (SLNs) are colloidal submicron particles made of lipids. They are biodegradable, readily scalable drug delivery systems with a high drug-to-fat ratio, which enables poorly soluble substances to become more soluble. SLNs can increase the solubility of natural Cur and decrease the lipopolysaccharide (LPS)-induced activity of pro-inflammatory mediators, such as NO, PGE2, and IL-6, by blocking NF-κB activation (Ganugula et al., 2017). Zhang *et al.* created Cur loaded nanoparticles (Cur-PEG-PDLLA) and assessed their impact on palmitate-induced cardiomyocyte death. It was observed that Cur-PEG-PDLLA reduced cardiomyocyte death, decreased Bax (a crucial mitochondria target), and increased Bcl-2 (an antiapoptotic protein that prevents Bax from oligomerizing) (Zhang et al., 2019). Therefore, the authors hypothesized that Bcl-2/Bax ratio modulation might be associated with the cardioprotective effect of Cur-PEG-PDLLA. Furthermore, Cur-PEG-PDLLA-treated cardiomyocytes had decreased ROS generation. According to their later study, these effects were associated with the modulation of particular downstream protein expression and the activation of the AMP-mediated protein kinase signaling pathway (Salehi et al., 2020).

### 4.4 Metallic nanoparticles of curcumin

**FIGURE 4:**
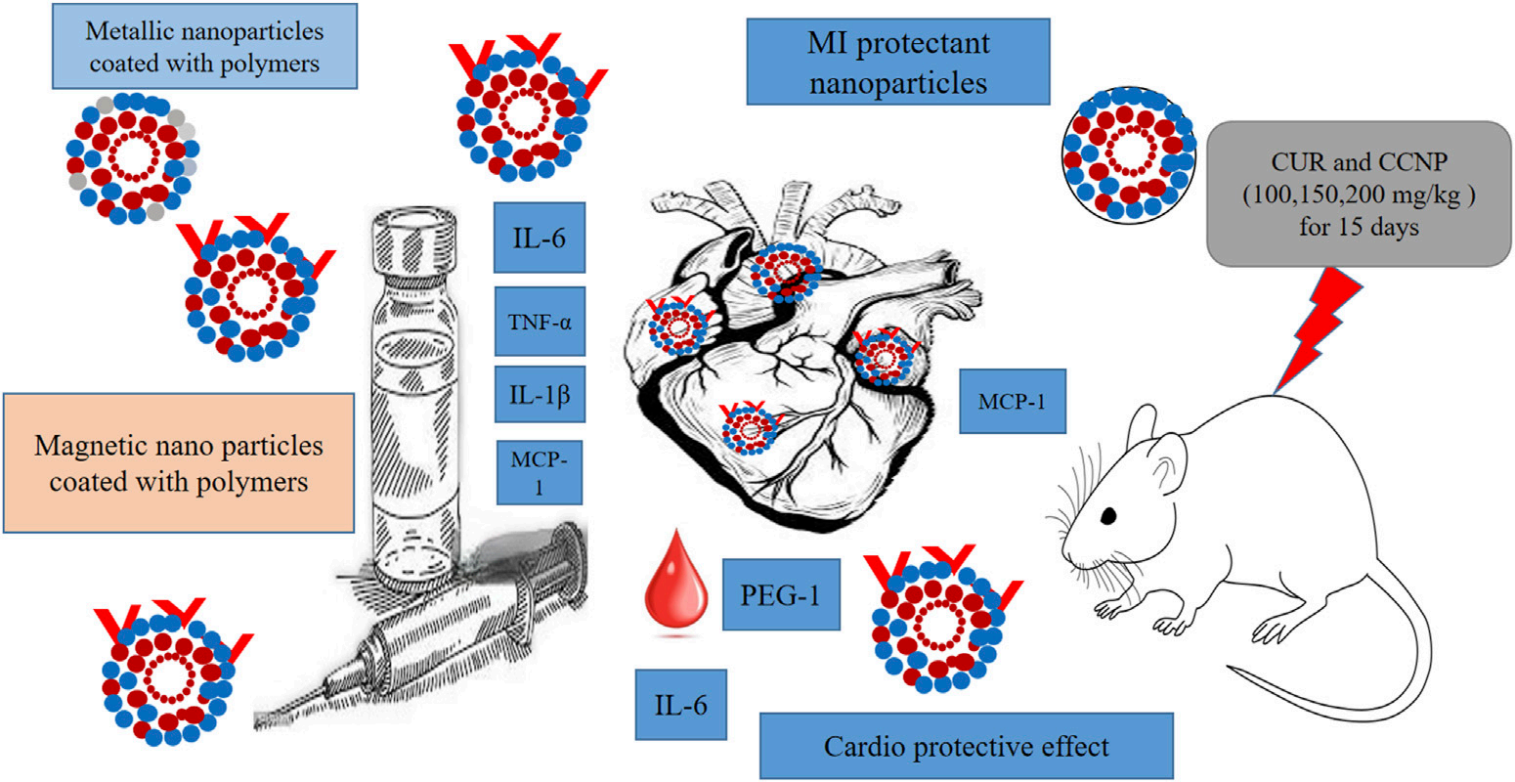
Illustration of the effects of metallic nanoparticles on MI. The visual elements represent the nanoparticle characteristics, *in-vivo* study details, and comparisons between different types of nanoparticles for MI detection.

Metallic nanoparticles (e.g., Fe, Zn, Ag, etc.) are inexpensive to prepare having special physical qualities. In a recent study, Lijuan Tan created a modern cardioprotective drug (eco-friendly, Zn NPs of Curcumin) to address the MI (iso-preterinol induced) in mice, focus on the PPAR-γ/NF-κB pathway (Tan, 2024). In mice with myocardial infarction, ZnNPs reduce cardiac inflammation. ZnNPs increase ventricular wall infarction, reduce mortality, and lower myocardial injury indicators. ZnNPs significantly attenuated the ST segment depression in mice with myocardial infarction. The mice with myocardial infarction in the pre + post-isoproterenol group showed more cardioprotective benefits from ZnNPs than those in the post-isoproterenol group. ZnNPs significantly reduced cell death and inflammatory cytokine expression *in vitro*. the results showed that ZnNPs may have favorable effects by normalizing gene expression for PPAR-γ/NF-κB/IκB-α/IKKα/β and phosphorylating PPAR-γ. This study found that ZnNPs protect against isoproterenol-induced myocardial infarction may be due to PPAR-γ activation and NF-κB signaling inhibition. In another recent study, Bourang et al. constructed iron oxide (Fe_3_O_4_) nanoparticles of cur and assessed them for colorectal cancer treatment *in vitro* (Bourang et al., 2024). Their study showed that the nanoparticles had high cytotoxicity on colorectal cancer cells and little damage on non-cancerous cells, indicating a well-targeted drug delivery strategy for future colorectal cancer treatments, suggesting the safety of use in other conditions. Moreover, natural compound-coated nanoparticles with magnetic properties have demonstrated their effectiveness in CVD imaging. Suzuki *et al.* established an animal model *via* arterial thrombus and elastase-induced vascular damage. They reported that ultra-small superparamagnetic iron oxide nanoparticles were more effective than specific polysaccharides-coated MRI materials. Another example includes effective detection of MI using gold nanoparticles (AuNPs) as contrast agents. The technique relies on collagen-conjugated designed AuNPs, which have been shown to detect myocardial and ischemic damage with great resolution and provide appropriate therapeutic tool (Suzuki et al., 2015). Figure 4 illustrates the effects of metallic nanoparticles on myocardial infarction (MI).

### 4.5 Curcumin conjugates

Curcumin oral bioavailability and solubility can be enhanced by conjugating with small molecules and hydrophilic polymers. According to Manju and Sreenivasan, conjugating curcumin with hyaluronic acid enhances its water solubility and stability as well as reduces the impact of the effect of AuNPs (Manju and Sreenivasan, 2011). A conjugate of Cur to a food-derived hydrophilic hydroxyethyl starch (HES) with an acid labile ester linker was used in a study to create amphiphilic conjugates (Chen et al., 2020). The conjugate’s self-assembly produced homogenous micellar nanoparticles (HES-CUR NPs) with significant colloidal and storage stability, an acid-responsive release mechanism, and a favorable drug loading efficacy (Xu et al., 2018). Moreover, this nanoparticle enhanced Cur solubility hundreds of times than its free form and protected the loaded curcumin from UV radiation-induced deterioration. In addition, the *in vitro* cytotoxicity assays and radical scavenging investigations have indicated that HES-Cur NP’s increased solubility, stability, and bioavailability, considerably increased Cur cytocompatibility, anticancer, and antioxidant activity. Therefore, HES-Cur NPs can be used for creating functional food products or drug formulations that prevent or treat various illnesses, including cancer and inflammatory disorders (Wichitnithad et al., 2011).

### 4.6 Solid dispersions of curcumin

Solid dispersion is the molecular dispersion of two different substances (Ford, 1986). The solid dispersions are dissolved as tiny colloidal particles in an aqueous medium to release the medication (Okonogi et al., 1999). Solid dispersions can be made using solvent-based, fusion-melt techniques as well as hybrid techniques that combine the two. Li *et al.* established a curcumin-Eudragit^®^ PO solid dispersion *via* a solution mixing method to improve curcumin’s aqueous solubility and stability. Moreover, an *in vitro* transdermal study validated Cur@EPO’s suitability as a Cur delivery system for medical applications. In addition, several studies revealed spray-dried CUR-Gelucire^®^50/13 solid dispersion increased (3,600-fold) water solubility compared with the native Cur (Li et al., 2015).

### 4.7 Development of micelles curcumin nanoparticles

A micelle comprises amphiphilic surfactants that form a spherical vesicle when dissolved in a solvent. Micelle is commonly used as a drug delivery system for poor water soluble substances like curcumin (Gong et al., 2013). A recent clinical trial indicated that coronary angioplasty patients had improved lipid profiles and oxidative and inflammatory indicators after Cur nano-micelle treatment (80 mg/day), compared to Cur alone (500 mg/day) (Helli et al., 2021). Furthermore, curcumin nano-formulation significantly reduced the levels of TNF-α, triacylglycerol (TG), malondialdehyde, and SOD (Huang et al., 2019). Another study revealed that hemodialysis patients had reduced proinflammatory adhesion molecules, VCAM-1 and ICAM-1, as well as serum CRP levels after nano-Cur treatment (120 mg/day) (Vafadar afshar et al., 2020). Curcumin nano-micelle also significantly improved the levels of TG in patients with metabolic syndrome but failed to ameliorate other biochemical parameters such as TC, LDL-C, HDL-C, fasting blood sugar (FBS), hemoglobin A1c (HbA1c), homeostatic model assessment (HOMA) for insulin resistance (HOMA-IR), and pancreatic β cell function (HOMA-β) (Bateni et al., 2021). The research revealed that curcumin co-delivered with resveratrol (Cur-Res-mP127) significantly enhanced its aqueous solubility, approximately 1617-fold more than the solubility of the free Cur. Experimental results demonstrated that Cur-Res-mP127 provided cardio protection in rat embryonic cardiomyocytes (H9C2) treated with doxorubicin hydrochloride by reducing apoptosis and ROS (Carlson et al., 2014).

Increase in L-type Ca2+ channel-mediated ROS production and ischemia-reperfusion damage can cause CVD. Scientists assessed the effectiveness of poly (glycidyl methacrylate) nanoparticles enclosed Cur in (Cur-PGMA) and their amalgamation with a peptide that focuses on the α-interacting domain of L-type Ca2+ channels (Cur-AID-PGMA) in rat hearts that were exposed to ischemia-reperfusion. This study aimed to validate these formulations reduced the negative impact of ischemia-reperfusion by inhibiting ROS production and Ca2+ influx (Hardy et al., 2015). The average Cur-AID-PGMA nanoparticles diameter was 152 nm, and PDI was 0.062, suggesting a limited size availability. Cur loading efficiency in the nanoparticles was 11.8% (w/w). It was observed that after ischemia-reperfusion injury, both the Cur-PGMA and Cur-AID-PGMA formulations exerted protective effects against oxidative stress and myocardial injury, indicating their potential therapeutic utility in treating ischemia-reperfusion injury. Based on these results, a study comprehensively investigated the effectiveness of these formulations and highlighted that Cur-loaded nanoparticles reduced myocardial damage and targeted oxidative stress, thereby mitigating the negative effects of ischemia-reperfusion injury (Mohamadian et al., 2022). These data provide the basis for the possible clinical use of Cur-based nanoparticles for the treatment of heart conditions. Ray et al. (2016) showed that while keeping curcumin’s bioactivity, its encapsulation in carboxymethyl chitosan nanoparticles (Cur-CMC), bioavailability is enhanced. Furthermore, Cur-CMC suppressed the heart hypertrophy in rat model’s. Similar to free Cur nano formulation enabled the observation of advantageous effects at a low dose (5 mg/kg body weight).

### 4.8 Nanospheres and microcapsules

Microcapsules have an exterior polymeric shell and an internal core. S Manna *et al.* synthesized selenium nanoparticles encapsulating poly (lactide-co-glycolide) nanospheres using Cur and assessed them against Alzheimer’s disease, suggesting the use of this carrier system for cur delivery (Huo et al., 2019). Hydrophilic drug carriers with a positively charged surface are important for increasing Cur permeability across biological barriers such as the blood-brain barrier. Recently, a cationic polymer, N, N, N-Trimethyl Chitosan (TMC), which is a quaternized chitosan derivative, was established. The TMC synthesis was validated *via* infrared spectroscopy (FTIR) and nuclear magnetic resonance (1H-NMR). A nano-emulsion technique was employed to synthesize TMC-based nano-spheres for loading Cur (Manna et al., 2023). These studies suggest the use of such curcumin formulations in cardiovascular diseases like MI.

Natural antioxidants in the human diet, such as Cur and resveratrol are employed to treat and prevent various oxidative stress-related illnesses. Lipid-core nano-capsules comprising Cur and resveratrol were prepared to enhance the antioxidant properties of Cur and resveratrol. Physical and chemical properties were assessed and compared with formulations comprising each polyphenol separately. The nano-technological properties of all formulations showed a mean diameter of approximately 200 nm, a low PDI, and nearly 100% encapsulation efficiency without any significant impact from co-encapsulation. It was also observed that resveratrol and Cur were more photo-stable when nanoencapsulated, whereas resveratrol indicated higher photostability when co-encapsulated. The *in vitro* antioxidant activity of polyphenols against OH radical was enhanced by nanoencapsulation, and a better effect was observed after their co-nano encapsulation. Moreover, both nanocapsulated polyphenols indicated a regulated release profile. Overall, it was observed that co-encapsulating Cur and resveratrol can enhance their therapeutic and preventive efficacy against oxidative stress-related illnesses (Coradini et al., 2014).

### 4.9 Piperine nanoformulations as a cofactor for MI therapy

Nano-formulations of piperine as a cofactor for MI therapy represent a promising approach to improve the bioavailability and therapeutic efficacy of bioactive compounds (Tagde et al., 2021). It significantly improves curcumin’s pharmacokinetics and absorption and overcomes its poor bioavailability, facilitating delivery to ischemic myocardial tissue. The administration of This synergy may decrease oxidative stress, inflammation, and apoptosis and promote cardiomyocyte survival and tissue repair (Tagde et al., 2021). Piperine has also been found to promote angiogenesis and, therefore, support tissue regeneration. Several studies have used the co-delivery strategy of Piperine and curcumin for enhacing the curcumin’s bioavailability with good results (Moorthi et al., 2012; Tu et al., 2014; Baspinar et al., 2018; Ahmadi et al., 2023). These nanoformulations, however, present challenges in ensuring stability, targeted delivery, and safe long term effects, which will need further experimental and clinical investigation before clinical application (Wang Z. et al., 2023).

### 4.10 Local infusion strategies

In recent years, injectable hydrogel systems have significantly progressed in biological applications. These biomaterials offer numerous benefits of targeted administration, controlled release, and improved mechanical qualities (Caillaud et al., 2018). Researchers assessed if a magnetic hydrogel nanocomposite loaded with Cur could effectively treat heart hypertrophy. They induced cardiac failure in 10 rats by administering 2.5 mg/kg of doxorubicin for 2 weeks. The rats weighed between 150 and 200 g. Then, Cur-loaded magnetic hydrogel nanocomposite was administered to the test groups, whereas the control group was only administered with Cur (Namdari and Eatemadi, 2017). After 2 weeks of the last dosage, the levels of MDA and GPX enzymes were observed. Furthermore, the expression of three heart failure markers, including beta major histology complex (β-MHC), B-type natriuretic peptide (BNP), and atrial natriuretic peptide (ANP), was examined. The expression of these markers decreased as Cur concentration increased (*p < 0.05*). In cardiac tissue, Cur decreased the raised level of MDA and increased the levels of SOD and GPX. Altogether, it was inferred that this combination could be employed to treat hypertension and heart failure (Namdari and Eatemadi, 2017). These studies suggest the use of local administration of curcumin could significantly enhance its therapeutic potentials in MI. Table 2 shows some prominent studies of curcumin in cardiovascular diseases.

**TABLE 2:** Curcumin-based nano-formulations for cardiovascular diseases.

Curcumin nano-formulations	Induction models	Result	References
Egg phosphatidylcholine liposomes coated with curcumin	*In vivo*	Demonstrated ischemia-reperfusion damage cardioprotection	Rogers et al. (2012)
Liposomes nanoformulations	*In vivo*	Indicated liposomes are particularly made to deliver atorvastatin calcium and curcumin for the management of atherosclerosis	Li et al. (2019)
Curcumin nanomicelle	*In vivo*	A clinical experiment revealed that patients receiving coronary angioplasty experience improved lipid profiles as well as oxidative and inflammatory indicators after taking curcumin nano micelle (80 mg/day)	Helli et al. (2021)
Curcumin nano-emulsion	*In vivo* rat model	Indicated that curcumin nano-emulsion increased the inactivation of HMGR to demonstrate its antihypercholesterolemic effect	Rachmawati et al. (2016)
Curcumin Nanoparticles	*In vivo* rat Model	This study used the cardiomyocytes to thoroughly examine the impact of curcumin nanotechnology on the generation of reactive oxygen species, which are associated with NADPH oxidase, cardiac apoptosis, and the modification of protein signaling pathways	Li et al. (2017)

## 5 Clinical trials of nano-curcumin formulation for cardia-vascular diseases

Cur is the most investigated polyphenol in CVD treatments because it inhibits the enzyme activity of p300 histone acetyltransferase, a gene associated with heart failure. Theracurmin^®^ is commercially available nano-technology of highly accessible Cur (Imaizumi, 2015). It comprises 10% Cur, 2% additional curcuminoids, 4% gum ghatti, and 84% water. A clinical trial was conducted by administering low and high (150 and 210 mg, respectively) Theracurmin^®^ dosages administered in healthy people to assess dose-dependent plasma Cur levels (Kanai et al., 2012). The study’s results demonstrated that, without overloading the absorption system, nano-technology raises plasma Cur levels in a dose-dependent manner. Moreover, Doppler technology echocardiography of hypertensive patients from different clinical studies has indicated that Theracurmin (60 mg/day) for 24 weeks enhances variables of diastolic function. These results indicate that nanotechnology enhances left ventricular diastolic function in hypertensive patients without affecting blood pressure (Kanai et al., 2012). Although various studies have investigated polyphenol nanoformulations, their clinical has several limitations. This might be because polyphenol concentrations required for humans are far lower than those required *in vitro* or *in vivo* small animal models for beneficial effects. In addition, maintenance of the bioavailability of the bioactive components is critical to the efficacy of nano-nutraceutical products (Imaizumi, 2015).

### 5.1 Recent progress in nano-technology-based curcumin formulations

Recent advances in nanomedicine have significantly facilitated drug delivery, diagnosis, and treatment, especially for neurological, cardiovascular, and cancerous conditions. Targeted medication delivery is improved by stimuli-responsive nanoparticles and surface-modified nanocarriers, which release drugs in response to specific stimuli or deliver them directly to diseased tissues (Hesari et al., 2021). Nanoparticles have transformed mRNA and CRISPR delivery in gene therapy, increasing efficacy and safety. Nanotechnology has significantly improved liquid biopsies and immediate diagnostics, enabling quicker and more precise disease diagnosis. Bio-degradable nanoparticles reduce toxicity, while personalized nanomedicine tailored therapies to individual genetic profiles. Advances in immune nanomedicine like nanorobotics offer the potential for transformative minimally invasive treatments (Scafa Udriște et al., 2024). Rachmawati *et al.* employed 3-hydroxy-3-methylglutaryl coenzyme A reductase (HMGR) and acetylcholinesterase (ACE) assay kits to evaluate the antihypertensive and anti-hypercholesterolemic properties of Cur nano-emulsion *in vitro*. They revealed that nano-emulsified Cur inhibit ACE more than pure Cur (Rachmawati et al., 2016). When Cur/P was administered *in vivo*, cardiac cells’ serum levels of H2S and Ca2+ content increased, and their expression of the calcium-sensing receptor, calmodulin, and cystathionine-γ-lyase was upregulated. Moreover, Cur and Cur/P alleviated the pathological and morphological loss of cardiac cells and substantially reduced diabetic cardiomyopathy. Two Cur nano-formulations were studied against ischemia-reperfusion injury in rat hearts: 1) Cur-PGMA and 2) Cur combined with a peptide targeting the α-interacting domain of L-type Ca2+ channel (Cur-AIDPGMA). It was observed that both these nano-formulations were effective against ischemia-reperfusion-induced oxidative stress and myocardial injury (Ray et al., 2016). CCNPs (50 mg/kg) were employed for treating monocrotaline-induced pulmonary arterial hypertension in the rat model. It was revealed that CCNP treatment attenuated the development of right ventricular hypertrophy, decreased right ventricle weight to body weight ratio, decreased right ventricle mRNA expression of TNF-α and IL-1β, and diminished oxidative stress (Boarescu et al., 2019b).

A study utilized isoproterenol hydrochloride (ISO) to induce MI in rats and then investigated the effects of the administration of nano cur (Nc) and CUR solutions. Pre- and post-ISO treatment rats indicated that Cur and Nc did not affect the diastolic and systolic blood pressures. Before being treated with ISO, rats that received varied of Cur and Nc showed a decrease in heart rate and extended RR interval. However, how Cur lowered heart rate remains undetermined. The highest dose of Cur and Nc (200 mg/kg/body weight) was more effective for cardioprotection (Boarescu et al., 2019b). Lipid peroxidation, nitric oxide, myocardial MDA levels, and overall oxidative state decreased after Cur and Nc pretreatment. Rats that received Nc pretreatment indicated reduced nitric oxide levels, whereas Cur and Nc-treated ISO-induced rats had reduced blood LDH, ALT, and AST levels. However, Nc had more potential effects (Boarescu et al., 2019b). Hybrid Cur lipopolymer nanoparticles are also employed as a QT prolongation preventive measure (Ranjan et al., 2013). Boarescu *et al.* indicated that pretreatment with Cur and Nc decreased necrosis, myofibrillar degeneration, interstitial edema, and significant cardiac damage to tissue. Therefore, it was recommended that Nc should be administered to inhibit the progression of cardiac tissue damage in cases of acute MI (Boarescu et al., 2019b).

An antimicrobial peptide called nisin reduced the number of squamous carcinomas of the head and neck cells while inducing an arrest of the cell cycle and apoptosis (Joo et al., 2012). In mammals, Cur-nisin nanoparticles (CurNisNp) have been observed to be absorbed well without any toxicity. The preliminary treatment of ISO-induced guinea rats with CurNisNp before MI resulted in elevated ECG patterns and decreased ROS and MDA levels, serum myeloperoxidase activity, and cardiac troponin I. Cur enhanced the reduced transverse striations, necrosis, myofibril thickness, and hypertrophic index (Nabofa et al., 2018). Moreover, a novel drug delivery system of Curcumin was prepared and its administration significantly increased rats’ cardiac output and reduced their posterior wall thickness (Yadav et al., 2019; Sunagawa et al., 2012).

Rats were used to test the effectiveness of Cur-loaded mesoporous silica nanoparticles (MSNs) against doxorubicin-induced cardiac toxicity. The preliminary Cur-MSNs treatment reduced MDA levels but increased SOD and catalase levels in cardiac cells, thereby protecting the heart from the deleterious effects of recurrent doxorubicin delivery. The cardiac tissue histology of Cur, MSNs, and doxorubicin-treated patients indicated enhanced myofibrils and cytoplasm vacuolization. Moreover, the cardioprotective effects of Cur-MSNs might be because of their anti-inflammatory and antioxidant abilities, as well as their increased bioavailability (Yadav et al., 2019). Liu *et al.* found that cardiomyocytes exposed to CCNPs had lower levels of intracellular ROS, Rac1 activation, and cell death. Moreover, NP treatment also reduced the expression of palmitate-induced gp91phox, inhibited ROS production, and enhanced SOD activity or suppressed NADPH isoforms. In addition, it stopped apoptosis. It was determined that stress-associated inflammation of the endoplasmic signaling system inhibits NADPH-induced oxidative stress. Therefore, CCNPs were proposed as a potential formulation to ameliorate cardiac harm caused by lipid toxicity. CCNPs may promote these effects by modulating the expression of particular proteins and initiating the adenosine monophosphate-activated protein kinase signaling pathway (Liu et al., 2019). The impact of a magnetic hydrogel composite loaded with doxorubicin-induced MI in rats was also studied. Cur-NIPAAM-MAA-NP’s reduced the expression of the heart failure indicators β-myosin heavy-chain gene, B-type natriuretic peptide, and atrial natriuretic peptide, thereby validating its cardioprotective efficacy (Daya et al., 2022). In addition, the injection of lipid nano-emulsions loaded with Cur (CmLN) and coupled with a nano-arginine peptide (R9CmLN) indicated little cytotoxic and hemolytic effects on human endothelial cells (Simion et al., 2016). A recent investigation revealed that PLGA-CCNPs can increase the oral bioavailability of Cur and decrease liver fat accumulation, thereby improving cardiovascular responses. Another study indicated that CCNPs stopped cardiomyocyte CK-MB leakage in an ISO-induced MI rat. After MI induction, no increase in the circulation levels of IL-1α, MCP-1, IL-6, IL-1β, and TNF-α was observed. In a study using rats, the injection of PLGA nanoparticles co-loaded with gold and capped with Cur prevented heart hypertrophy by enhancing bioavailability, solubility, and absorption, as well as by resisting enzymatic breakdown and medication delivery effectiveness (Hesari et al., 2021). In addition, the antioxidant, antiapoptotic, and anti-inflammatory properties of CAu-PLGA Nps improved survival rates, maintained cardiac output, and protected rodents from cardiac toxicity and heart failure (Xie et al., 2011). These studies suggest that nanoformulations of curcumin could offer more safer and better therapeutic option for cardiovascular ailments like MI.

## 6 Conclusion and future perspectives

Curcumin has been a promising therapeutic agent due to its anti-inflammatory, antioxidant and cardioprotective characteristics. It has extensive preclinical and clinical evidence of its ability to alter key pathological CVD processes such as hyperlipidemia, atherosclerosis, and ischemic injury. However, its poor bioavailability as a function of dosage and formulation is limited by clinical utility. The recent development of Cur loaded nanoparticles like liposomes, polymeric nanoparticles and solid lipid nanoparticles has been made possible by recent advancements in nanotechnology. These Nano formulations enhance its bioavailability, provide improved cellular uptake, and more precise tissue targeting, greatly enhancing Cur’s therapeutic effects. Although these advances have been made, there are still many hurdles in clinical translation of Cur based nanomedicines including optimizing nanoparticle formulations, scalability of production, and feasibility.

The future of Cur nanomedicine should be focused on personalized approach to treat their nanomedicine according to individual genetic profiles, metabolic responses and co morbidities to improve the treatment efficacy. Further management of CVD may be possible by the integration of Cur with other therapeutic agents, including anti-inflammatory drugs or growth factors, that target multiple disease pathways. In addition, the design of nanotheranostics (which combine therapeutic and diagnostic functionalities within a single nanocarrier) promises the opportunity to optimize the delivery of Cur and monitor treatment progress on a real-time basis, thereby enhancing more personalized and dynamic treatment regimens. Preclinical data in support of the potential of Cur-loaded nanocarriers is strong, however, robust clinical trials are needed to confirm the safety, efficacy, and long-term effects of these formulations. Cur nanoformulations also require rigorous toxicological studies to assess safety profiles, especially for potential off-target effects. Additionally, cost-effective, scalable production methods will be required to translate these formulations into widespread clinical use.

## Abbreviations

Cur, curcumin; SIRT1, silent information regulator; SOD, superoxide dismutase; Nrf2, nuclear factor erythroid-derived; GSK-3β, glycogen synthase kinase-3B; PI3K, Phosphoinositide 3-kinase; ERK, extracellular signal-regulated kinase; ROS, reactive oxygen spices; IL-6, interleukin; HAPNPs, hydroxyapatite nanoparticles; CurNP, curcumin nanoparticles; CCNP, curcumin nanoparticles, HES, hydrophilic hydroxyethyl starch; CRP, C reactive protein; PGMA, poly (glycidyl methacrylate); PLGA, poly (lactide-co-glycolide); MDA, malondialdehyde; ANP, atrial natriuretic peptide; MI, myocardial infarction; JNK, c-Jun NH2-terminal protein kinase; TNF-α, tumor necrosis factor α; CVD, cardiovascular diseases; PDI, polydispersity index; GPX, glutathione peroxidase.
